# Dynamic and Postural Changes in Forelimb Amputee Dogs: A Pilot Study

**DOI:** 10.3390/ani14131960

**Published:** 2024-07-02

**Authors:** Oliver Rodriguez, Marta Regueiro-Purriños, Pedro Figueirinhas, José Manuel Gonzalo-Orden, Iván Prada, José Manuel Vilar, Lorena Millán, José Rodríguez-Altónaga

**Affiliations:** 1 Departamento de Patología Animal, Universidad de Las Palmas de Gran Canaria, Trasmontaña S/N, 35416 Arucas, Spain; oliver.rodriguez@ulpgc.es (O.R.); pedro.figueirinhas@fpct.ulpgc.es (P.F.); 2 Departamento de Medicina y Cirugia Veterinaria, Campus de Vegazana, Universidad de León, 24071 León, Spain; mregf@unileon.es (M.R.-P.); jmgono@unileon.es (J.M.G.-O.); vetivi@hotmail.com (I.P.); lmilv@unileon.es (L.M.); jarodma@unileon.es (J.R.-A.)

**Keywords:** limb amputation, force, pressure, dog

## Abstract

**Simple Summary:**

It is assumed that dogs with an amputated limb experience difficulty with their tripedal gait in terms of energy and balance. This is especially true when the missing limb is a forelimb. The aim of our study is to objectively deepen the knowledge about biomechanical (postural and dynamic) modifications in forelimb amputee dogs by using force and pressure platforms. Based on our results, the amputee dogs in our study had increased vertical, braking and propulsion forces and their respective impulses on their remaining limb also increased, except for the propulsion impulse during walking. The amputee dogs also had increased peak pressure, mean pressure and area of the paw. Surprisingly, the amputee dogs were able to preserve the same level of balance when compared with the control, four-legged dogs of the same breed at stance. Although amputee dogs were able to preserve balance during stance and gait, there was a higher force (and pressure) demand on the remaining forelimb. This situation may potentially predispose these animals to injury caused by an overload of the anatomical structures involved in weight bearing.

**Abstract:**

The amputation of a limb in quadrupeds can overload the remaining limbs, especially the contralateral one. The compensatory effort is especially high if it is a forelimb. It is, therefore, important to objectively know the changes in weight redistribution that occur in the animal while walking and standing still. With this objective, static (postural) and dynamic kinetic examinations were carried out on five French bulldogs with an amputated forelimb and five intact French bulldogs. For this examination, force and pressure platforms were used. The results were statistically compared using the student *t*-test. The parameters derived from the ground reaction forces were significantly higher in the amputee group. Surprisingly, postural examination showed that amputated dogs reached the same stability as healthy ones. Tripedal support in dogs does not objectively imply a loss of balance in quantitative terms; although the increase in force used by the remaining limb, as well as its altered cranial disposition during the support phase, may potentially predispose the animal to additional injuries in the future due to an overuse of different musculoskeletal units.

## 1. Introduction

Limb amputation due to an injury or as a consequence of a salvage surgical procedure compromises a quadruped’s locomotor performance and affects the functioning of the whole musculoskeletal system. Therefore, amputee dogs develop compensatory mechanisms in order to walk without losing balance [1,2,3]. Nevertheless, and in general terms, when a dog’s fore or hindlimb has been amputated, an excellent functional recovery has been reported [4,5,6], although the posture and mobility of amputee dogs are modified in most instances [7,8]

Nevertheless, there is little scientific literature objectively reporting on the changes in a dog’s gait and kinetic variations due to the redistribution of force [9]. Specifically, kinetic variations in forelimb amputee dogs often provide a classic peak vertical force (PVF) and vertical impulse (VI) [10,11]; however, no references regarding postural exams in amputee dogs could be found by this research team. In order to fully understand the musculoskeletal adaptation of the tripedal gait, it is necessary to complement the abovementioned PVF and VI with new, additional parameters. It is also crucial that we understand how these functional adaptations alter the normal dynamics of musculoskeletal units (tendons, muscles, ligaments, joints…) to better understand possible clinical consequences.

For instance, the remaining forelimb carries more weight after a forelimb amputation than a remaining hindlimb would after a hindlimb amputation [10], which means that forelimb amputees are more prone to suffer injuries resulting from weight overload; specifically, after the amputation of a forelimb, at the level of the contralateral limb’s triceps brachii and deltoid muscles, the load bearing increases, leading to fatigue and potential overuse injuries. In addition, the latissimus dorsi and erector spinae muscles of the trunk and spine have to work harder to stabilize and move the body, often resulting in back pain and muscle fatigue. But muscular and articular consequences are also suffered by the biceps femoris, quadriceps femoris and gluteus medios muscles of the hindlimb because of their increased strain to aid in locomotion and balance [2,12,13,14,15]. For this reason, it is necessary to understand the specific biomechanical adaptations in forelimb amputated dogs [4,6,12]. It was hypothesized that the amputation would change not only the magnitude of the vertical GRFs, but also the horizontal plane and their corresponding impulses. If this scenario were true, it would also increase the load on the remaining limbs, especially the contralateral limbs, and move the dog’s center of gravity away from the site of amputation [10].

Using the basic principles of physics, the center of pressure (COP) is assumed to be the vertical projection of the center of gravity over the plane of support [16]. Modifications of the COP are currently measured with pressure platforms; these devices utilize multiple sensors to study the COP sway in sound and lame dogs, both at walk and at standing [17,18]. In this sense, our hypothesis is that forelimb amputee dogs alter a number of parameters at walk and even when standing still, and these changes could be objectively assessed.

The aim of this paper is to objectively detect and measure the dynamic (gait) and static (postural) changes in single forelimb amputee French bulldogs when compared with sound dogs of the same breed and conformation. With this aim, different parameters (vertical and horizontal forces and impulses, COP pathway, statokinesiogram, etc.) will be obtained by using force and pressure platforms.

## 2. Materials and Methods

### 2.1. Animals

A total of 10 adult, client-owned French bulldogs were enrolled in this retrospective, controlled study. A total of 5 dogs had a single forelimb amputation, and the other 5 dogs were sound and served as the control group. Both groups had 2 male and 3 female dogs.

#### 2.1.1. Amputee Group

The 5 dogs comprising the amputee group had their forelimbs amputated for the following reasons: sarcoma (*n* = 3) and trauma (*n* = 2). These dogs were clinical cases from the Hospital Clinico Veterinario of the University of Las Palmas de Gran Canaria from February 2021 to June 2023 and were retrospectively included in this study. All animals received standard forelimb amputation including scapulectomy. Dogs unable to walk comfortably or that had any orthopedic and/or neurological abnormal findings in any of the remaining limbs on previous clinical examination performed 1 week before the force and pressure platform analyses were excluded from the study. These analyses were performed a minimum of 4 and a maximum of 7 months post amputation. Age and weight ranged from 5 to 8 years and 9.5 to 14 kg, respectively.

#### 2.1.2. Control Group

All 5 dogs comprising the control group were healthy, sound animals which came to our hospital for their annual routine examination and vaccination. These animals did not have current or previous history of orthopedic and/or neurological disease. Age and weight ranged from 6 to 9 years and 10 to 12.5 kg, respectively.

All dogs belonging to both groups received a score of 5–6 on the Body Condition Score (BCS) scale from the Association for Pet Obesity Prevention (https://www.petobesityprevention.org/ accessed on 10 May 2024).

### 2.2. Force and Pressure Platform Analysis

The design of the study for the acquisition of force- and pressure-related parameters has been developed in agreement with previous studies [19,20].

#### 2.2.1. Force Platform Analysis

**Figure 1:**
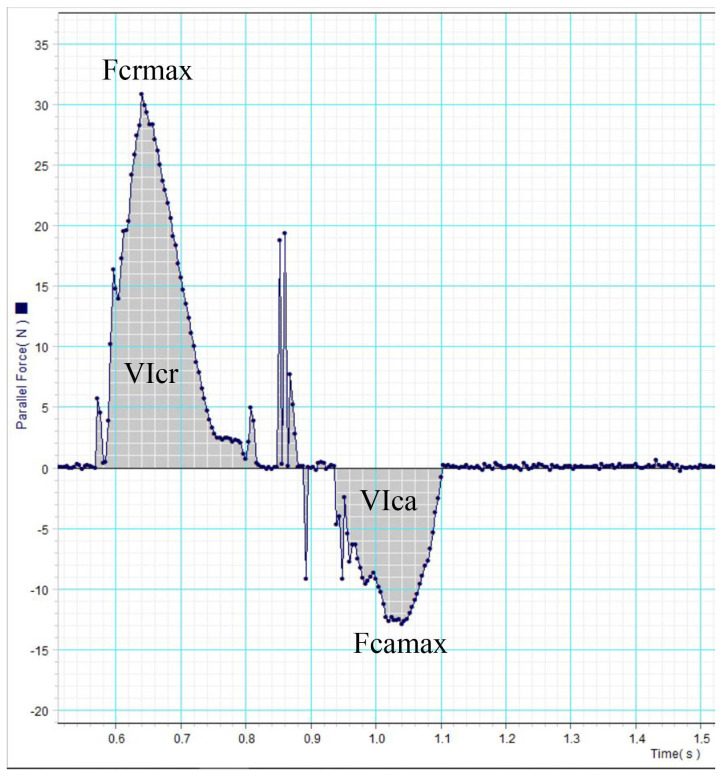
Force platform recording of an amputee dog. Horizontal forces and impulses are represented.

The force platform (Pasco, Roseville, CA, USA) consisted of a dynamometric, 4-sensor force platform of 35 × 35 cm and a sample frequency of 250 Hz. The device was placed in a 10 m long corridor. The platform was inserted into a purpose-built hole in such a way that the device’s surface was level with the ground and was covered with a rubber mat. Specific software (DataStudio® version 1.9.8r10, Pasco, Roseville, CA, USA) was used to obtain peak vertical (Fvmax), cranial or braking (Fcrmax) and caudal or propulsive (Fcamax) forces (N) from three valid trials (Figure 1). Walk velocity was measured with a motion sensor (Pasco, Roseville, CA, USA) positioned 1 m from the platform. A trial was considered valid when the dog’s walking speed was within the range of 0.5–0.7 ± 0.2 m/s, no movement of the limbs, head and/or neck was observed and the handler did not have any physical contact with and/or did not restrain the animal during the recording (Video S1). Mean values were normalized to body weight (%BW). Vertical impulses (N*s) for the vertical, cranial and caudal directions were also obtained (VIv, VIcr and VIca, respectively) (Figure 1).

#### 2.2.2. Pressure Platform Analysis

**Figure 2:**
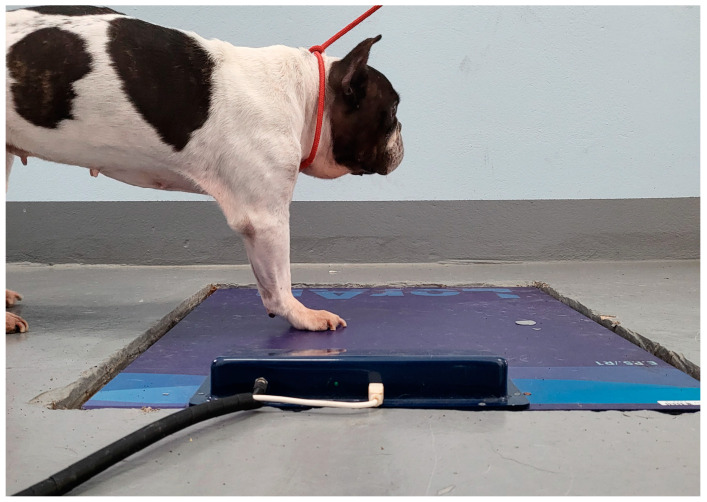
Postural exam of an amputee dog. Note that the leash around the neck is loose.

A pressure platform (EPS/R1, Loran Engineering, Bologna, Italy) with Biomech software version 1.6.1.14687 (Loran Engineering, Bologna, Italy) was used, consisting of 2096 pressure sensors (density 1 sensor/cm^2^) evenly spread over a quadrangular frame. The acquisition frequency was 100 Hz, and the range of pressure measured was 30–400 Kpa. The platform was placed in another purpose-built cavity level with the floor and adjacent to the force platform in order to ensure that the recordings from the animals were obtained in the same trial. To perform the postural exam (posturography), the dogs were placed with both forelimbs on the platform. The dog remained standing still for at least 10 s (Figure 2). To ensure that the dog stayed in place, the owner was directly placed in front of the dog to ensure that the head and neck were facing forward without turning to the side. A total of three trials were carried out for each animal. The obtained posturographic data included statokinesiogram (Stat, mm^2^), peak and mean pressure (PP, MP, Kpa) and paw area (PA, cm^2^). To study the COP pathway characteristics, dogs were leash led and walked in the same way as they were during the force platform analysis.

As general criteria during the force and platform analyses and in order to obtain the most representative data possible for the statistical analysis, the mean value of the three trials was considered as long as the trials differed by <10%. When the difference was >10%, new trials were carried out to obtain three valid results.

### 2.3. Statistical Analysis

The Shapiro–Wilk test was used to assess the normality of the variables. In all cases, the Shapiro–Wilk test *p*-value was greater than 0.05, indicating that normality can be assumed for all variables. Therefore, the variables were summarized using the mean and standard deviation. The mean value of each variable was compared between sound and amputee dogs using Student’s *t*-test. The Holm correction was applied to adjust for multiple testing. Differences with a *p*-value less than 0.05 were considered statistically significant. The R Software and environment version 4.4.1 [21] was used to perform the statistical analysis.

## 3. Results

The mean (±SD) time from amputation to the beginning of the force and pressure platform analyses for the amputee group was 4.8 ± 1.3 months. The mean (±SD) ages of the amputee and control groups were 7 ± 1.6 and 7.4 ± 1.1 years, respectively. No statistical differences were found between the two groups (*p* = 0.66). The mean (±SD) weights of the amputee and control groups were 11.2 ± 1.8 and 11.9 ± 1.14 kg, respectively. No statistical differences were found between the two groups (*p* = 0.49).

The following table shows the mean ± SD and *p*-values of both groups (Table 1).

Regarding force values, Fvmax, Viv, Fcrmax and VIcr were significantly higher in the amputee group, while Fcamax and VIca did not significantly differ.

**Figure 3:**
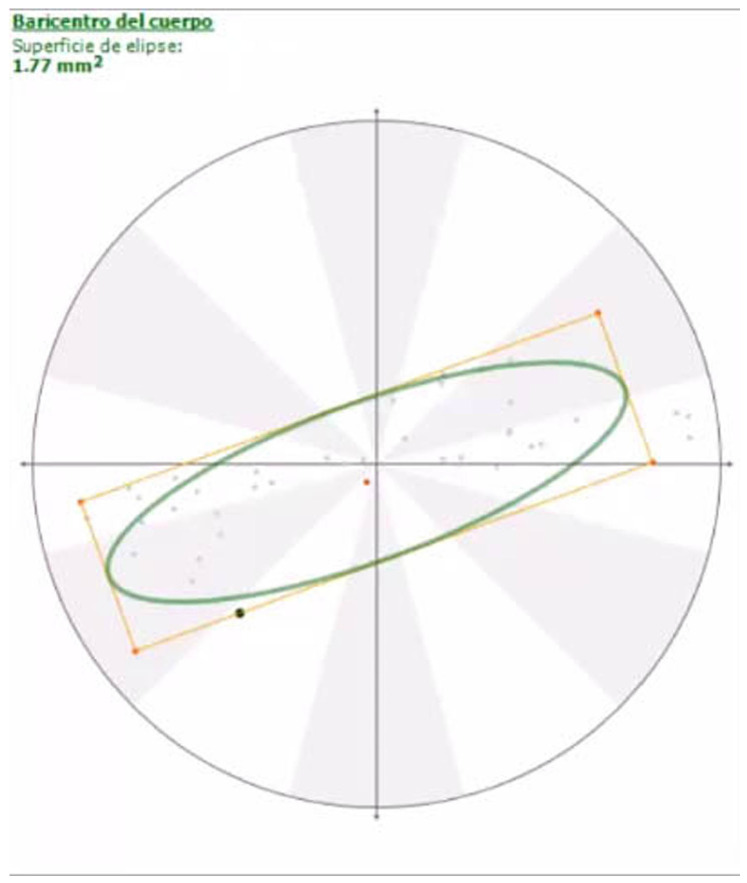
Statokinesiogram of a sound dog. Note the laterolateral orientation of the ellipse.

**Figure 4:**
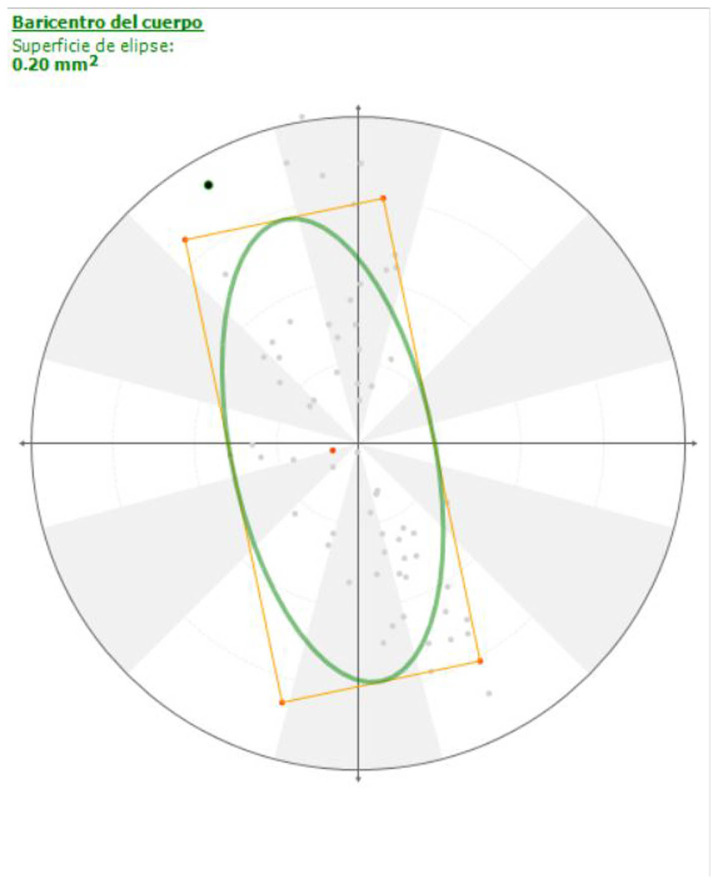
Statokinesiogram of an amputee dog. Note the craniocaudal orientation of the ellipse.

For the pressure data, the area of the statokinesiogram showed no differences between the amputee and sound dogs, although the orientation of the ellipse changed from horizontal to sagittal (Figure 3 and Figure 4; Videos S2 and S3); other static data, such as PP, MP and PA, were significantly higher in the amputee group.

**Figure 5:**
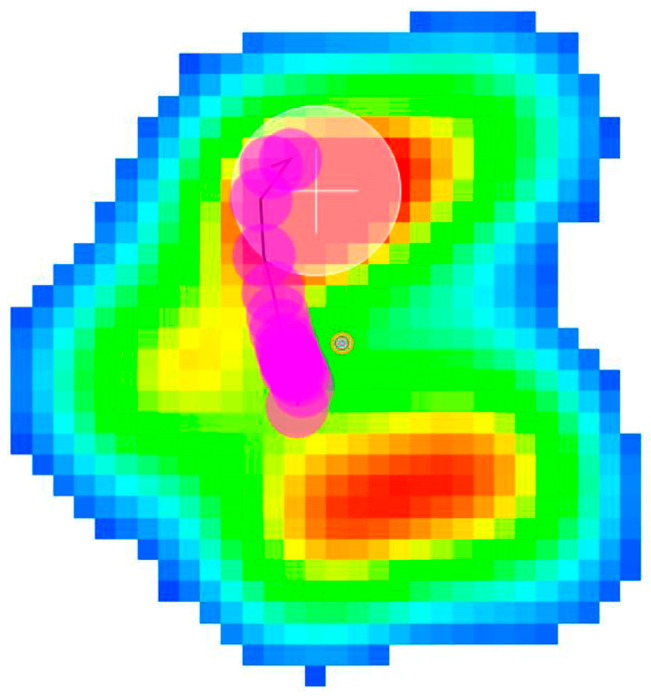
Paw pressure recorded during the support phase of a sound dog. The consecutive pink dots represent the COP pathway. The top of the image is cranial, bottom is caudal, right is medial and left is lateral. Note that the pathway starts at the center of the paw and runs cranially.

**Figure 6:**
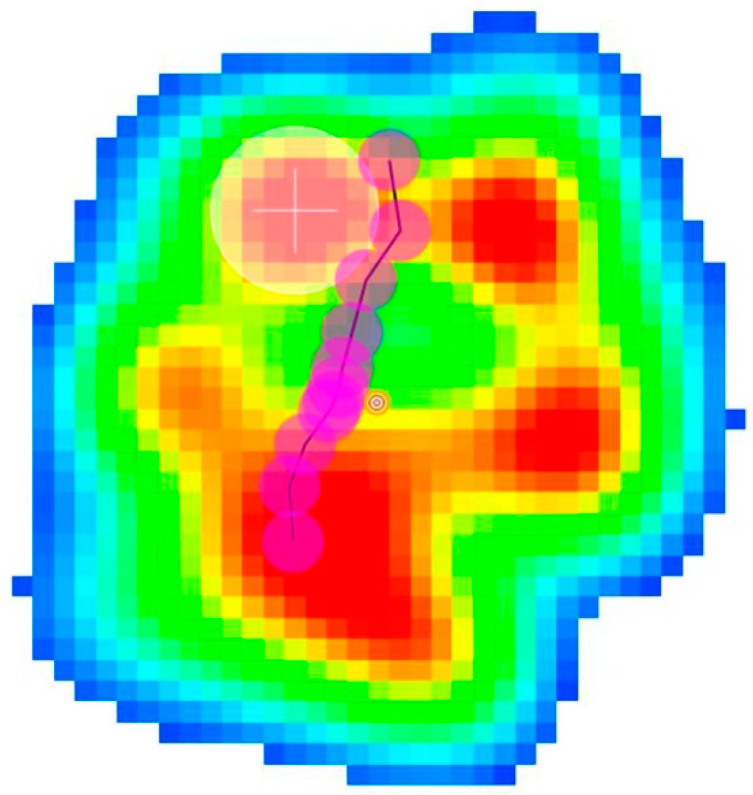
Paw pressure recorded during support phase of an amputee dog. The consecutive pink dots represent the COP pathway. The top of the image is cranial, bottom is caudal, right is medial and left is lateral Note that the pathway starts more caudally at the level of the metacarpal pad and runs cranially; this proves that when the limb makes contact with the ground, it is placed more cranially than sound forelimbs.

Additionally, beyond the numerical data, the study of the COP pathway in the animals at walk revealed that sound dogs begin contact with the ground approximately at the center of the paw and this pathway runs cranially during the support phase of the gait. However, the amputee dog’s COP came into contact with the ground more caudally, at the level of the metacarpal cushion (Figure 5 and Figure 6; Videos S4 and S5).

## 4. Discussion

The present study compared the results obtained for a list of kinetic parameters using force and pressure platforms between a group of five French bulldogs with a forelimb amputated and a group of five sound dogs of the same breed.

Prior to the biomechanical assessment, the study needed to ensure that the amputated dogs were fully adapted to the tripedal gait. Previous studies reported that most dogs are adapted within a month [3], especially if the amputation concerns the forelimbs [4,5,11]. The recovery time for normal locomotive activity may differ due to different intrinsic factors, such as age, body weight and breed [22,23,24,25,26]. In our case, all the dogs were totally accustomed to the tripedal gait, given that amputation had been performed at least 4 months prior in all cases.

Dog speed and weight may alter kinetic parameters [27]; for this reason, special efforts have to be made in order to obtain reliable data to be able to make comparisons between homogeneous groups. Regarding speed, it was maintained in a narrow range to be considered “valid” as described in the methods section; on the other hand, and given that the amputee dogs were already undergoing a weight control program to avoid overloading the remaining limb, the same criteria were applied to control dogs in order to maintain them within the “ideal” weight for the breed.

The presence of a residual stump in non-complete limb amputations, such as proximal humeral osteotomies, can hinder the comparison with full-leg amputees, since in this situation the animal has the option of using it to partially maintain correct balance; this was not the case in our study because all our animals underwent full-leg amputation.

As shown in the results, almost all of the force parameters (Fvmax, Fcrmax) and their respective impulses (VIv, VIcr) showed higher values, which clearly proves that there was a net redistribution of weight to the remaining forelimb. This occurs not only in terms of force, but also in terms of the duration of the braking phase, which means that amputee dogs spend more time in this phase than sound dogs, as found in previous studies [10]. On the contrary, the Fcamax and VIca values remained the same, meaning that the propulsion force and duration did not change in the amputee group. We believe that this occurs because while the force during the braking phase has to be “assumed” by the remaining forelimb, the contribution of the hindlimbs during the propulsion phase is very important, as noted by other authors; this inverse relationship between force and phase duration has been previously published [11,28,29,30].

Regarding postural analysis, it has been suggested that dogs with an amputated forelimb tend to have more difficulty maintaining their balance [31,32]; however, our postural results obtained from the statokinesiograms showed that forelimb amputees maintain the same level of balance. In other words, tripedal support is as effective as quadrupedal support in terms of balance.

Previous stance studies showed that the ellipse remains with a transversal orientation in both sound and lame dogs due to dogs having a greater stability in the sagittal plane because the longitudinal axis is longer than the horizontal axis. In our study, the fact that the ellipse orientation changed to a sagittal plane was surprising because it meant that the COP pathway axis changed from transversal to primarily parallel to the dog’s longitudinal axis, although the dog’s balance did not change.

As occurred with changes in force, the amputee dogs also applied more pressure when their limbs came into contact with the ground, which proves that the weight redistribution at walk changes compared to dogs with a sound forelimb in cases of painful lameness [20] or, as in our case, when a forelimb has been amputated. Paw expansion is a consequence of the pressure exerted by the limb on the ground given the elastic nature of the digital and metacarpal cushions. Our results showed a clear increase in paw surface in amputee dogs, although this expansion does not seem to be proportional to the increase in pressure [33].

Finally, the limb COP pathway moved more caudally in the amputee group, which proves that the remaining forelimb is located more cranially than that in sound dogs at the beginning of the support. We believe that the increased force and pressure values, as well as alterations in limb placement during the support phase, leads to there being a greater amount stress on the remaining forelimb, especially on the structures that contribute to the body’s support.

All of the increases in the force and pressure values shown here make the animals more predisposed to overuse injuries, as said in the introduction. In our opinion, preventive measures should be taken; among them, rehabilitation and physiotherapy such as hydrotherapy, flexibility training and weight control as well as environmental adaptations such as the avoidance of slippery surfaces and the use of ramps instead of stairs generally facilitate safe ambulation [13,14,15].

The results shown here contribute to the knowledge of dynamic and postural adaptations in dogs who have experienced a forelimb amputation. However, this study has some limitations: first, the study used a relatively low number of animals; although the fact that all of the animals used in this study are the same breed, and with the same BCS (thus, of the same morphotype), allowed for more homogeneous and reliable data and increases the strength of the conclusions, as reported in previous studies based on force platform analysis [34]. Second, weight redistribution to the hindlimbs in forelimb amputee dogs has been reported, but this fact was not considered in our study. This could be investigated in future research.

## 5. Conclusions

Tripedal support in dogs does not objectively imply a loss of balance in quantitative terms. However, there is an increase in the amount of force used by the remaining limbs and an altered disposition of the limbs during the support phase. These facts may potentially predispose an animal to additional injuries in the future due to the overuse of different musculoskeletal units.

## Figures and Tables

**Table 1:** Mean ± SD and *p*-values of both groups. Asterisk (*) means significant difference.

Force Values	Sound	Amputee	*p*-Value
Fvmax	99.35 ± 3.63	182.77 ± 10.59	0.0018 *
VIv	15.86 ± 1.81	28.06 ± 0.58	0.0028 *
Fcrmax	9.60 ± 1.68	25.31 ± 0.75	0.0004 *
VIcr	0.74 ± 0.31	2.49 ± 0.15	0.0028 *
Fcamax	5.21 ± 0.87	12.11 ± 2.21	0.0740
VIca	1.00 ± 0.20	1.16 ± 0.16	0.5672
Stat	0.23 ± 0.03	0.23 ± 0.05	0.9267
PP	215.8 ± 5.07	292.4 ± 10.16	0.0180 *
MP	78 ± 0.71	82.50 ± 3.26	0.0740
PA	14.50 ± 2.38	20.00 ± 1.00	0.0632

Units: Fvmax, Fcrmax, Fcamax: N (%BW); VIv, VIcr, VIca: N.s (%BW); Stat: mm^2^; PP, MP: KPa; PA: cm^2^.

## Data Availability

Data are contained within the article and Supplementary Materials.

## Supplementary Materials

The following supporting information can be downloaded at: https://www.mdpi.com/article/10.3390/ani14131960/s1, Video S1: High speed video showing a force platform trial. Video S2: Statokinesiogram of a sound dog. Video S3: Statokinesiogram of an amputee dog. Video S4: COP pathway of a sound dog. Video S5: COP pathway of an amputee dog.
